# Inulin accelerates weight loss in obese mice by regulating gut microbiota and serum metabolites

**DOI:** 10.3389/fnut.2022.980382

**Published:** 2022-09-28

**Authors:** Zeang Wu, Zhenzhu Du, Yuanyuan Tian, Miao Liu, Kailong Zhu, Yufan Zhao, Haixia Wang

**Affiliations:** ^1^First Affiliated Hospital, School of Medicine, Shihezi University, Shihezi, China; ^2^Analysis and Testing Center, Shihezi University, Shihezi, China; ^3^School of Medicine, Shihezi University, Shihezi, China

**Keywords:** obesity, inulin, gut microbiota, *Alistipes*, indole-3-acrylic acid

## Abstract

Several studies indicated that the gut microbiota might participate in the beneficial effect of inulin on obesity. However, the mechanisms involved were still largely unknown. Sixteen high-fat diets (HFDs)-induced obese C57BL/6 mice were converted to a normal diet and then randomized into two groups, OND (obese mice + normal diet) group gavage-fed for 10 weeks with normal saline and ONDI (obese mice + normal diet + inulin) group with inulin at 10 g/kg/day. The body weight of HFD-induced obese mice showed different degrees of decrease in both groups. However, the ONDI group lost more weight and returned to normal earlier. Compared to the OND group, inulin supplementation significantly shifted the composition and structure of gut microbiota, such as higher α diversity. The β diversity analysis also confirmed the changes in gut microbiota composition between groups. At the genus level, the abundance of *Alistipes* was considerably increased, and it was significantly correlated with inulin supplementation (*r* = 0.72, *P* = 0.002). Serum metabolite levels were distinctly altered after inulin supplementation, and 143 metabolites were significantly altered in the ONDI group. Among them, indole-3-acrylic acid level increased more than 500-fold compared to the OND group. It was also strongly positive correlation with *Alistipes* (*r* = 0.72, *P* = 0.002) and inulin supplementation (*r* = 0.99, *P* = 9.2e−13) and negatively correlated with obesity (*r* = −0.72, *P* = 0.002). In conclusion, inulin supplementation could accelerate body weight loss in obese mice by increasing *Alistipes* and indole-3-acrylic acid level.

## Introduction

Currently, public health is threatened by the increasing occurrence of obesity worldwide. It is reported that 20% of the adult population will be subjected to obesity by 2030 (1). Obesity is caused by a complex interplay of genetic and environmental factors and is thought to result from a chronic imbalance between energy intake and expenditure (2). Excessive consumption of high-fat diets (HFDs) can increase adipose tissue (3), predispose to obesity (4), and induce metabolic and cardiovascular disorders (5). Meanwhile, a HFD can wreak havoc on the balance of gut microbiota in the host (6–8). Clinical studies have found that gut microbiota dysbiosis is closely related to obesity (9, 10), which can affect the occurrence and development of obesity by regulating lipid metabolism and energy homeostasis (11). Growing evidence has shown that prebiotics, especially inulin, can alleviate glucose, lipid metabolism disorders, and obesity by modulating gut microbiota (12–15).

Inulin is a common prebiotic, defined as an indigestible dietary fiber that is beneficial for the growth of probiotics (16). The European Food Safety Authority has identified only one prebiotic: inulin improves intestinal function (17). It has been widely reported that inulin could alter the gut microbiota of obese individuals, increase the abundance of *bifidobacteria* and *Akkermansia muciniphila* in obese individuals, and improve metabolic disorders (18–22). Furthermore, inulin supplementation may promote short-chain fatty acid (SCFA) production in overweight or obese men (12). Animal experiments have also shown that inulin supplementation was associated with changes in the colon SCFAs levels, including acetic acid, propionic acid, and butyric acid (14). Dietary supplementation of the SCFAs was shown to significantly inhibit the body weight gain by enhancing triglyceride hydrolysis and FFA oxidation in the adipose tissue, promoting beige adipogenesis and mitochondrial biogenesis, and inhibiting chronic inflammation (23). Therefore, the effect of inulin on weight loss may be related to the interaction between gut microbiota and related metabolites.

However, alterations in metabolite levels were not limited to SCFAs, and there may be many other inulin-related metabolites that were not well studied. The development of non-targeted metabolomics has allowed us to understand better the impact of a factor on the overall metabolic profile of the host to find new biomarkers. In this study, we sought to unravel the important role of inulin in regulating gut microbiota and related metabolites and in accelerating the weight loss of HFD-induced obese mice, and further investigate whether the therapeutic effect of inulin on obesity is associated with gut microbiota and metabolites. Therefore, we examined changes in body weight, gut microbiota composition in feces, and metabolites in serum.

## Materials and methods

### Animals and study design

**FIGURE 1:**
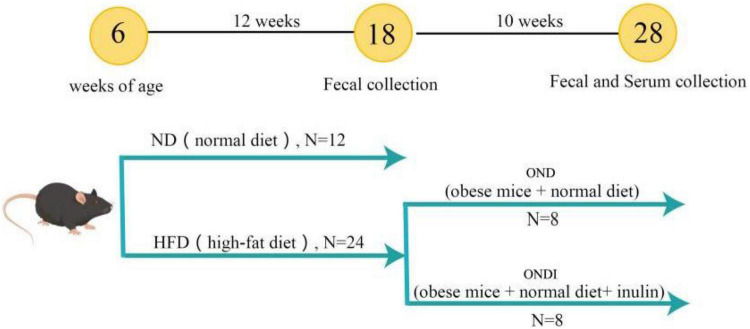
Graphical illustration of experimental design. C57BL/6J male mice were fed a normal diet (ND) and a high-fat diet (HFD) for 12 weeks to establish an obesity model. Subsequently, the diet of the obese mice in the HFD group was switched to a normal diet and supplemented with inulin.

In total, 36 male 4-week-old C57BL/6 mice were purchased from the Experimental Animal Center of Xinjiang Medical University. Mice were kept in the environmental control room (25 ± 3°C, 40 ± 5% humidity, 12 h light/dark cycle) with free access to food and water. After 1 week of acclimatization, an experimental design was shown in Figure 1. All mice were randomly divided into two groups and treated for 12 consecutive weeks. The first group was fed a normal diet (ND, Kcal%: 10% fat, 20% protein, and 70% carbohydrate; 3.85 kcal/gm, *n* = 12). The second group was fed a HFD (Kcal%: 60% fat, 20% protein, and 20% carbohydrate; 5.24 kcal/gm, *n* = 24). After the obesity model of mice in the HFD group was established successfully, the surviving mice in the HFD group were randomly subdivided into two groups (*n* = 8 per group) that were fed a normal diet for 10 weeks. Specifically, (1) obese mice + normal diet (OND) group, obese mice were fed with a normal diet; (2) obese mice + normal diet + inulin (ONDI) group, obese mice were fed with a normal diet with inulin (Cosucra Co., Ltd., Belgium) at 10 g/kg/day by gavage. All the mice had *ad libitum* access to diet and water. During the period of treatment, the body weight and food intake were recorded weekly. By the end of the experiment, the mice were sacrificed after 12 h of fasting. Orbital blood sampling was used to collect plasma of mice in each group, and the feces was also collected for the follow-up experiments, immediately frozen in liquid nitrogen, and stored at −80°C. White adipose tissue (epididymal, retroperitoneal, and perirenal fat) and liver were weighed.

### Gut microbiota analysis

Genomic DNA was extracted from fecal samples using a TIANamp Stool DNA kit (TIAN GEN Bio-Tech Co., Ltd., Beijing, China). The variable V3–V4 region of the bacterial 16S rRNA gene was amplified by PCR using primers (F 5′-ACTCCTACGGGAGGCAGCAG-3′ and R 5′-GGACTACHVGGGTWTCTAAT-3′). 16S rRNA amplicons were detected on the Illumina NovaSeq platform by Novogene (Beijing, China). Paired-end reads from sequencing were merged utilizing Fast Length Adjustment of SHort reads (FLASH) (24) to obtain raw tags. Then use fastp software to carry out quality control on the obtained raw tags to obtain high-quality clean tags. Finally, the Usearch software is used to compare the clean tags with the database to detect and remove chimeras, so as to obtain the final effective tags. For the above-obtained effective tags, use the DADA2 module in the QIIME2 software to denoise, and filter out sequences with an abundance of less than 5, so as to obtain the final Amplicon Sequence Variants (ASVs). QIIME2’s class-sklearn algorithm (25, 26) was used for species annotation for each ASV using a pre-trained Naive Bayes classifier.

The alpha diversity (within-sample diversity) was assessed using Shannon, Simpson, Pielou, and Chao 1 indexes. Beta diversity (between-sample diversity) was measured with non-metric multidimensional scaling (NMDS) and principal coordinate analysis (PCoA). ANOSIM analysis with unifrac distance was applied to test for significant group clusters differences. Diagrams are visualized using R packages. The linear discriminant analysis effect size (LEfSe, LDA > 4) was applied to distinguish the vital bacterial biomarkers of the differential representation within groups by Qiime2. PICRUSt2 version 2.2.0 was used to predict the gut microbial metabolic functions based on the 16S sequences. The plot was performed on the Tutools platform,^1^ a free online data analysis website.

### Serum metabolite profiling

Detection of non-targeted metabolites in serum samples was based on liquid mass spectrometry (LC-MS) by Novogene (Beijing, China). Take 100 μl of the sample and put it in an EP tube, add 400 μl of 80% methanol aqueous solution. Then take a certain amount of supernatant and add mass spectrometry grade water to dilute to 53% methanol, centrifuge at 15,000*g*, 4°C for 20 min, collect the supernatant for LC-MS analysis. Quality Control (QC) samples will be controlled throughout the process of on-machine testing, namely, before, during, and after LC-MS/MS sampling. The first three QC before injection were used to monitor the instrument state and balance the chromatography-mass spectrometry system. The following three QC were used for segmental scanning, and the secondary spectrum obtained from the experimental sample was used for the characterization of metabolites. QC inserted in the middle of sample testing is used to evaluate the system stability during the whole experiment process and conduct data quality control analysis.

The original files obtained by mass spectrometry were imported into Compound Discoverer 3.1 (CD3.1) software for spectral processing and database search, and the qualitative and quantitative results of metabolites were obtained. Based on high-resolution mass spectrometry (HRMS) detection technology combined with the mzCloud database, mzVault database, and MassList database, the characteristic molecular peaks were matched and identified to reflect the total metabolite information to the maximum extent. The metabolite peak was extracted, and the peak area was relatively quantified by CD3.1 software. Then, the metabolite was identified by comparison with mzCloud, mzVault, and MassList databases. Finally, the final identification results retain metabolites with a Coefficient of Variance (CV) of less than 30% (27) in QC samples.

The variable importance for the projection (VIP) values of the metabolites was obtained by PLS-DA (diagram is visualized using R package). Differential metabolites were screened according to VIP > 1, | log_2_*^FC^*| > 1.5 and *P* < 0.05. R was used to visualize the top 30 differential metabolite levels heatmap.

### Statistical analyses

Statistical analyses were performed with SPSS 22.0 (USA). All data were expressed as the mean ± SD. A two-tailed Student’s *t*-test analyzed the differences between groups. The correlation was tested by Spearman correlation analyses. GraphPad Prism Version 8.0 (USA) and R were used for plotting. *P < 0.05, ^**^P < 0.01, and ^***^P < 0.001 were considered statistically significant.

## Results

### Inulin supplementation contributes to the treatment of obesity

**FIGURE 2:**
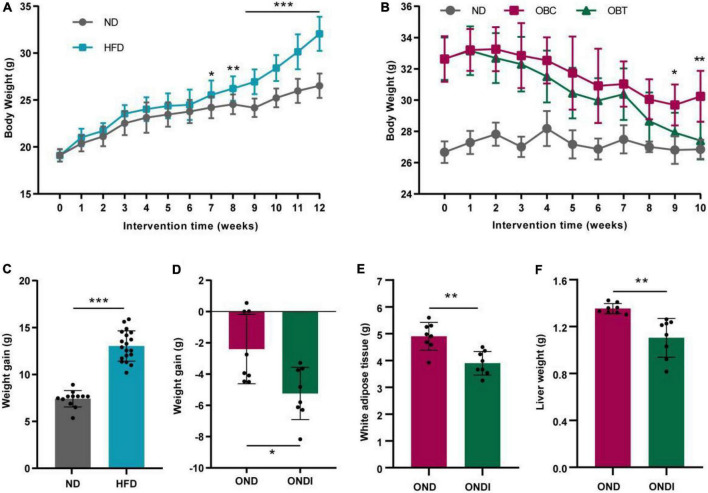
Effect of inulin on body weight, weight gain, white adipose tissue and liver weight. **(A)** Changes in body weight of mice fed with a high-fat diet for 12 weeks. **(B)** Changes in body weight of obese mice supplemented with inulin for 10 weeks. **(C)** Weight gain after 12 weeks of HFD treatment. **(D)** Weight gain after 10 weeks of inulin treatment. **(E,F)** White adipose tissue and liver weight after 10 weeks of inulin supplementation. Each value was expressed as mean + SD. *P < 0.05, **P < 0.01, and ***P < 0.001, HFD or OND compared with ND mice. ND, normal diet; HFD, high-fat diet; OND, obese mice + normal diet; ONDI, obese mice + normal diet + inulin.

After 12 weeks of HFD treatment, the final body weight and weight gain of mice in the HFD group significantly increased compared with ND group (Figures 2A,C). Subsequently, mice in the HFD group were switched to a normal diet for 10 weeks, and we found that both the OND group and ONDI group showed a tendency to lose weight. However, the weight loss of the ONDI group was faster, and the body weight in the ONDI group was significantly lower than that of the OND group from week 9. By the 10th week, compared with OND group the weight of the ONDI group first returned to normal, and the weight loss obviously increased, indicating that additional inulin supplementation accelerated weight loss in obese mice (Figures 2B,D). Besides, inulin supplementation did not significantly change food intake of mice in OND and ONDI groups (Supplementary Figure 1), the reduction of liver and white fat weight was closely related to the weight loss of ONDI group mice (Figures 2E,F).

### Effects of inulin on gut microbial diversity in obese mice

**FIGURE 3:**
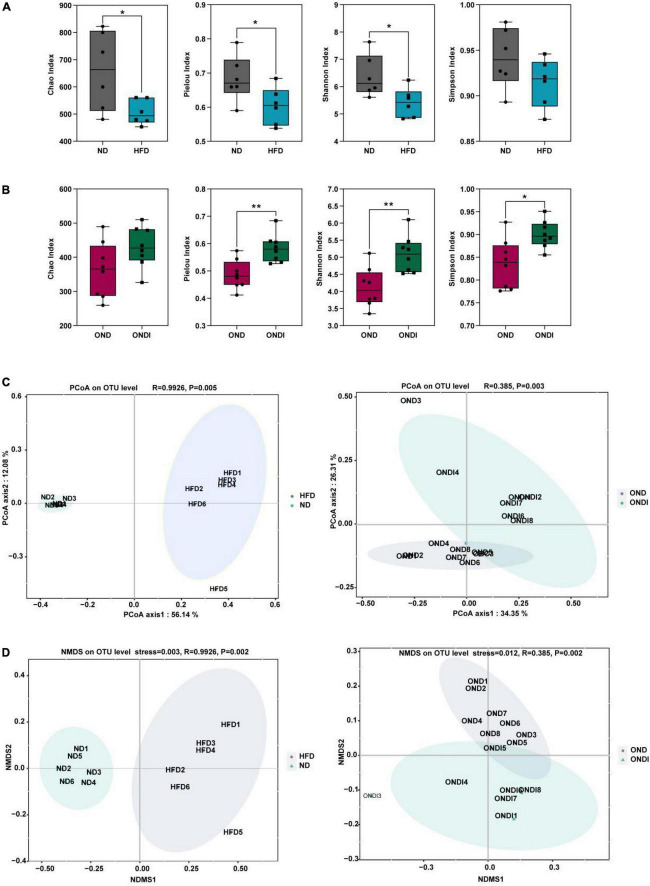
Effects of inulin supplementation on α and β diversity of gut microbiota in obese mice. **(A,B)** The Chao index accessed the community richness, and the community diversity was accessed by the Pielou, Shannon, and Simpson indices. **(C,D)** Principal coordinate analysis (PcoA) and NMDS analysis. Each value was expressed as mean + SD. *P < 0.05, **P < 0.01, ***P < 0.001. ND, normal diet; HFD, high-fat diet; OND, obese mice + normal diet; ONDI, obese mice + normal diet + inulin.

Compared with the ND group, high-fat feeding significantly reduced the α diversity, including reduced Chao index, Pielou index, and Shannon index considerably (Figure 3A). Compared with the OND group, inulin supplementation significantly increased the α diversity, such as the Pielou index, Shannon index, and Simpson index (Figure 3B). By using β diversity analysis based on PCoA and NMDS, the ND and HFD formed clusters separated from each other, indicating that gut microbiota composition changed significantly in response to HFD. Moreover, a similar phenomenon was also observed in the reaction to inulin supplementation between OND and ONDI groups (Figures 3C,D).

### Effects of inulin on differences of microbial composition in obese mice

**FIGURE 4:**
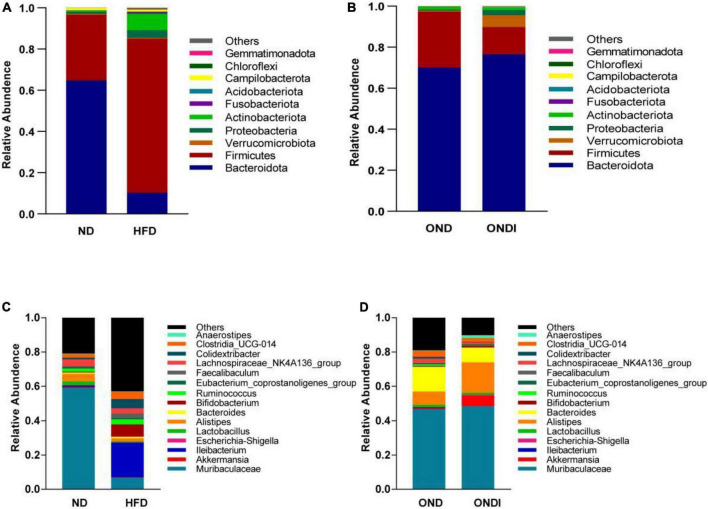
Relative abundance of the top 10 phylum-level **(A,B)** and genus-level top 15 **(C,D)** most important gut microbial components following inulin supplementation. Each column represents the composition of the microbial taxa in one group.

**FIGURE 5:**
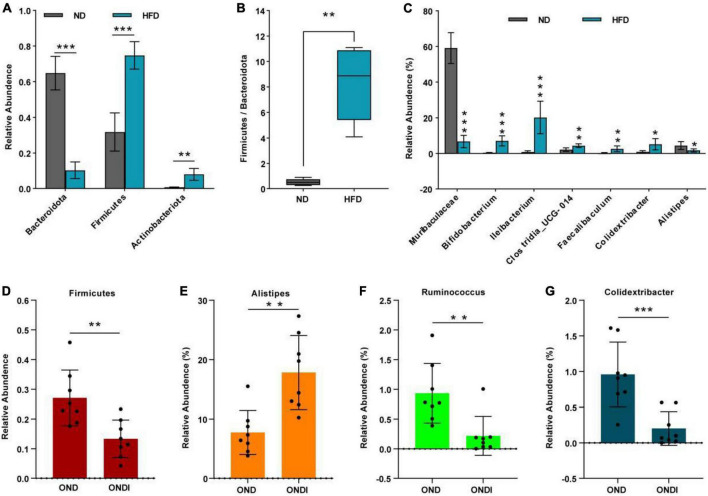
Alterations of feature gut species after inulin supplementation. **(A)** Changes in top 10 gut phyla between ND and HFD groups; **(B)** the ratio of the abundance of *Firmicutes* and *Bacteroidetes*; **(C)** changes in top 15 gut genus between ND and HFD groups. **(D)** Changes in top 10 gut phyla between OND and ONDI groups; **(E–G)** changes in top 15 gut genus between OND and ONDI groups. *P < 0.05, **P < 0.01, and ***P < 0.001.

Bar graphs were drawn according to the relative abundance of the top 10 most abundant gut bacterial phyla (Figures 4A,B) and the top 15 most abundant gut genera in different groups (Figures 4C,D). At the phylum level, *Firmicutes* and *Bacteroidetes* were the most abundant gut bacterial phyla, and their overall mean relative abundance of all groups was greater than 88%. Compared with the ND group, the abundance of *Firmicutes* and *Actinobacteriota* were obviously increased, while the abundance of *Bacteroidota* was significantly decreased in the HFD group (Figure 5A). Meanwhile, HFD feeding significantly increased the ratio of *Firmicutes* to *Bacteroidetes* (Figure 5B). Compared with the OND group, inulin supplementation significantly reduced the abundance of *Firmicutes* in the ONDI group (Figure 5D). At the genus level, high-fat feeding significantly decreased the abundance of *Muribaculaceae* and *Alistipes* and enriched *Bifidobacterium, Ileibacterium, Clostridia_UCG-014, Faecalibaculum*, and *Colidextribacter* compared with the ND group (Figure 5C). After supplementing with inulin, we found statistically significant changes in *Alistipes*, *Ruminococcus*, and *Colidextribacter* in the top 15 most important gut genera compared to the OND group. Notably, the relative abundance of *Alistipes* in the ONDI group was greatly increased to 17.8%, 2.3 times that of the OND group (Figure 5E). At the same time, *Ruminococcus* and *Colidextribacter* were significantly lowered (Figures 5F,G). Furthermore, *Alistipes* was strongly negatively correlated with *Colidextribacter* (*r* = −0.57, *P* = 0.023, Supplementary Figure 2A) and *Ruminococcus* (*r* = −0.55, *P* = 0.016, Supplementary Figure 2B), while *Ruminococcus* was strongly positively correlated with *Colidextribacter* (*r* = 0.86, *P* = 2.2e−05, Supplementary Figure 2C).

**FIGURE 6:**
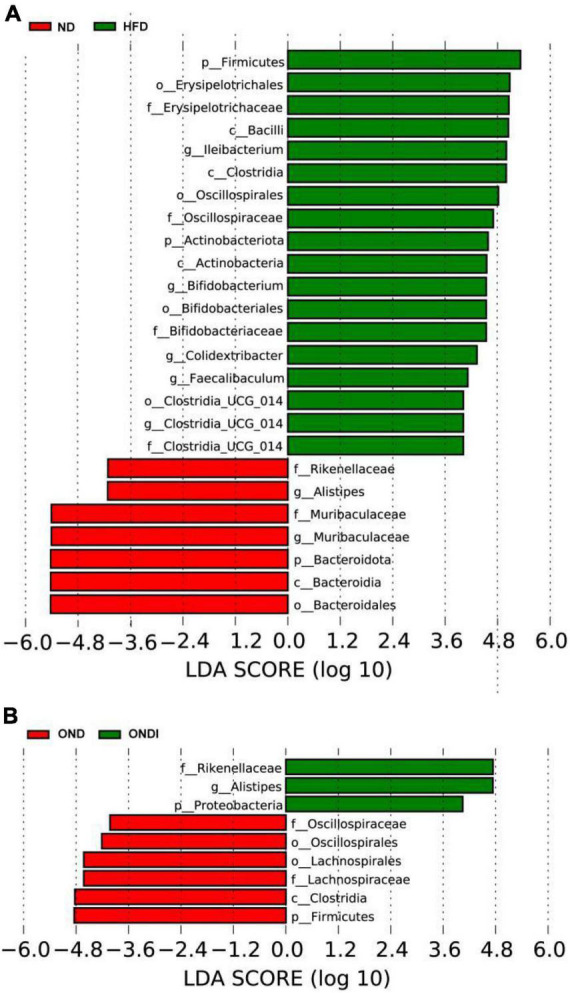
LEfSe analysis of key phylotypes of mice gut microbiota. **(A,B)** linear discriminant analysis (LDA > 4) scores derived from LEfSe analysis.

The LEfSe analysis showed that bacteria from phyla *Bacteroidota* (e.g., *Alistipes* genus and *Rikenellaceae* family) were significantly enriched in the ND group and the bacteria from the phyla *Firmicutes* were significantly associated with the HFD group. For example, *Ileibacterium, Colidextribacter, Faecalibaculum*, *Clostridia_UCG-014* genus from *Firmicutes* were mainly linked to the HFD group (Figure 6A). Surprisingly, inulin supplementation reversed the above phenomenon after 10 weeks. The *Alistipes* genus and *Rikenellaceae* family from phyla *Bacteroidota* especially distinguished the OND group from the ONDI group, while phyla *Firmicutes* were still significantly related to the OND group (Figure 6B). The cladogram corresponding to five phylogenetic levels (from phylum to genus) generated from LEfSe analysis showed the most relevant bacterial taxa among each group, consistent with the results mentioned above (Supplementary Figures 3A,B).

### Gut microbial metabolic functions

**FIGURE 7:**
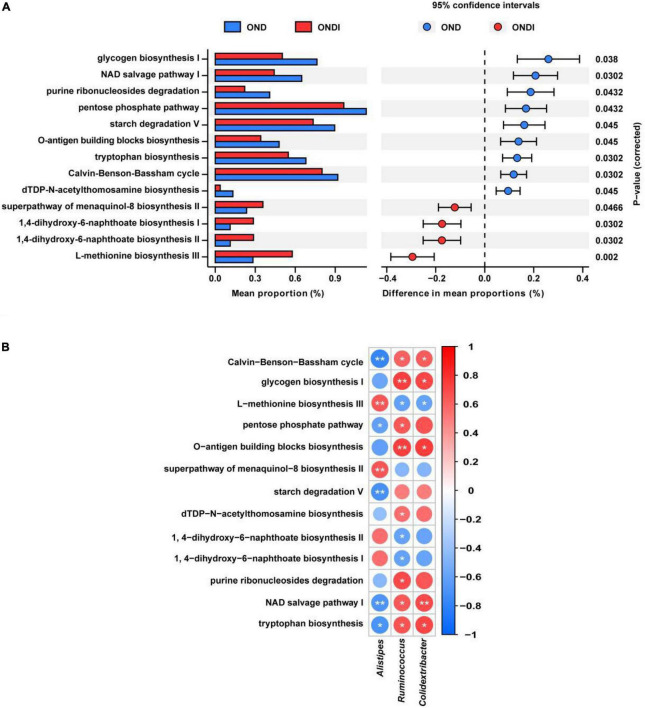
Predicted metabolic profile of the gut microbiota after inulin supplementation **(A)** and association of feature gut species and metabolic profile **(B)**. *P < 0.05, **P < 0.01.

PICRUSt2 predicted a total of 413 function pathways. According to the abundance distribution of each metabolic pathway in each sample, the metabolic characteristics of gut microbiota were predicted after inulin supplementation for 10 weeks. Compared with the OND group, inulin supplementation significantly reduced glycogen biosynthesis I, NAD salvage pathway I, purine ribonucleosides degradation, pentose phosphate pathway, starch degradation V, tryptophan biosynthesis, Calvin–Benson–Bassham cycle, and dTDP-N-acetylthomosamine biosynthesis; and enhanced superpathway of menaquinol-8 biosynthesis II, 1,4-dihydroxy-6-naphthoate biosynthesis I, 1,4-dihydroxy-6-naphthoate biosynthesis II and L-methionine biosynthesis III (Figure 7A). Furthermore, the three critical gut microbiota, *Alistipes*, *Ruminococcus*, and *Colidextribacter*, were statistically associated with these metabolic pathways. The *Alistipes* were significantly positively correlated with L-methionine biosynthesis III and significantly negatively correlated with tryptophan biosynthesis and NAD salvage pathway I, whereas the *Ruminococcus* and *Colidextribacter* were the opposite (Figure 7B).

### Effects of inulin on alterations of serum metabolites

**FIGURE 8:**
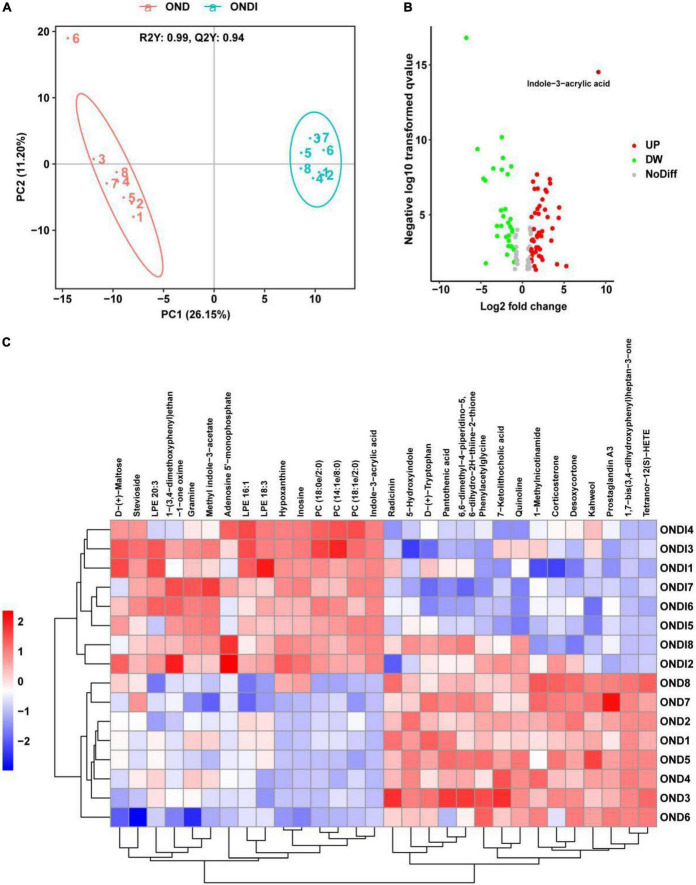
Serum metabolic profiles associated with inulin. **(A)** PLS-DA score plot showed clustering. **(B)** The volcano plot showed significant metabolites. **(C)** The heat map showed the top 30 metabolites clustering.

A total of 421 serum metabolites were detected using the positive ion mode of LC-MS. To identify the particular metabolites associated with inulin among thousands of variables, a pairwise comparison of the PLS-DA model was established with satisfactory validation (R^2^Y = 0.99, Q^2^Y = 0.94). There was a significant separation between the OND and ONDI groups (Figure 8A). According to the screening criteria: VIP > 1, *P* < 0.05, and | log_2_*^FC^*| > 1.5, a total of 113 differential metabolites were screened out, including 68 increased and 45 decreased metabolites (Figure 8B). The top 30 metabolites were selected to construct the heatmap (Figure 8C). The samples were well clustered into OND and ONDI groups, and the level changes of each metabolite within the group were basically consistent. The top 30 metabolites mainly include indoles and derivatives (indole-3-acrylic acid, tryptophan, 5-hydroxyindole, and methyl indole-3-acetate).

### Correlation analysis and regression analysis

**FIGURE 9:**
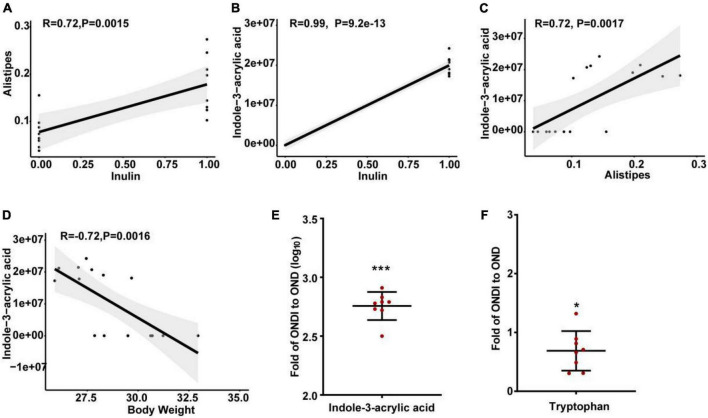
Spearman correlation analysis and changes in key metabolite levels after inulin supplementation. **(A)** The relationship between *Alistipes* and inulin; **(B)** the relationship between indole-3-acrylic acid and inulin; **(C)** the relationship between indole-3-acrylic acid and *Alistipes*; **(D)** the relationship between indole-3-acrylic acid and body weight; **(E,F)** indole-3-acrylic acid and tryptophan presented as the ratio of the abundance in ONDI mice to those in OND mice. *P < 0.05, ***P < 0.001.

The abundance of *Alistipes* in the feces of mice on a HFD was significantly reduced, and inulin supplementation after returning to a normal diet significantly increased the abundance of *Alistipes*. In addition, LEfSe analysis also showed that only the *Alistipes* appeared to have the most evident response to inulin at the genus level, so we further analyzed the correlation between *Alistipes* and inulin by Spearman correlation analysis. The abundance of *Alistipes* was shown to have a strong positive correlation with inulin (*r* = 0.72, *P* = 0.002, Figure 9A). Moreover, univariate linear regression analysis of *Alistipes* relative abundance (*y*) and inulin intervention (*x*) showed: *y* = 0.101*x* + 0.078 (*R*^2^ = 0.525, *P* = 0.001), indicating that inulin supplementation could significantly increase the relative abundance of *Alistipes*. Inulin treatment resulted in significant changes in metabolites, suggesting a certain association between metabolites and inulin. We found that the level of indole-3-acrylic acid was shown to have the strongest positive correlation with inulin among all the differential metabolites (*r* = 0.99, *P* = 9.2e−13, Figure 9B). Besides, it had a strong positive association with *Alistipes* (*r* = 0.72, *P* = 0.002, Figure 9C) and a strong negative association with body weight (*r* = −0.72, *P* = 0.002, Figure 9D). Surprisingly, we found that the level of indole-3-acrylic acid was most significantly increased by more than 500-fold among all differential metabolites after inulin supplementation compared with the NCD group (Figure 9E). Meanwhile, indole-3-acrylic acid with the largest VIP value in PLS-DA had the most obvious response to inulin. The indole-3-acrylic acid belongs to the indoles and is produced by the tryptophan metabolism. In our study, inulin supplementation significantly reduced tryptophan level compared with the OND group (Figure 9F).

## Discussion

Gut microbiota has been considered a key contributing factor in diet-related obesity. In our study, HFD disturbed gut microbiota balance and significantly increased the *F*/*B* value. Studies showed that the structure and composition of gut microbiota could be greatly affected by HFD (28), and obese individuals have relatively higher *F*/*B* values than normal-weight individuals (29), which is consistent with our findings. Inulin as food for probiotics can improve HFD-induced obesity and related metabolic disorders by modulating gut microbiota (13, 30). When obese mice switched from an HFD to a normal diet for 10 weeks, additional inulin supplementation led to faster weight loss and first return to normal weight compared with the OND group. In addition, the diversity of gut microbiota was reversed after inulin supplementation, the abundance of *Alistipes* was markedly increased, and the abundance of *Ruminococcus* and *Colidextribacter* was significantly decreased compared with the OND group. The *Alistipes* is a relatively new bacterial genus, mainly isolated from medical clinical samples and highly associated with dysbiosis and disease (31). A systematic review found that *Alistipes* was a lean-associated genus (32), and the HFD could obviously reduce the abundance of *Alistipes* (8, 33–36), whose abundance was inversely correlated to adiposity, serum lipids (including low-density lipoprotein, triglyceride, and total cholesterol), and glucose homeostasis parameters (37–39). Notably, the relative abundance of *Alistipes* in the ONDI group was significantly increased to 17.8%, 2.3 times higher than that of the OND group, and this might be one of the key reasons why inulin promotes rapid weight loss.

The abundance of *Ruminococcus* in obese individuals was higher than that in lean individuals (10), *Ruminococcus bromii* and *Ruminococcus obeum* had a significant correlation with obesity in the Japanese population (40). In our study, the abundance of *Ruminococcus* in the HFD group was higher than that in the ND group (no statistical difference). In contrast, the abundance of *Ruminococcus* obviously decreased after inulin treatment compared to the OND group. The *Colidextribacter* was considered a HFD-dependent taxa, and some functional foods (such as flavonoids from whole grain-oats and partially milled barley) could modulate the perturbation of *Colidextribacter* in HFD-induced mice (41, 42). Consistent with our findings, mice fed an HFD significantly increased the abundance of *Colidextribacter*, which was reversed by inulin treatment compared to the OND group. In addition, *Alistipes* showed a clear negative correlation with *Colidextribacter* and *Ruminococcus*, suggesting the significant increase in *Alistipes* abundance seems to have an inhibitory effect on *Colidextribacter* and *Ruminococcus* after inulin supplementation. Furthermore, the LEfSe analysis showed that only *Alistipes* had a significant biological association with the ONDI group at the genus level. These findings suggest that inulin-accelerated weight loss in obese mice may be associated with the reversal of gut microbiota disturbances, primarily mediated by *Alistipes*.

On the other hand, inulin supplementation changed the overall serum metabolite profile of obese mice. Inulin is known to mitigate the development of obesity by producing SCFAs, including acetic acid, propionic acid, and butyric acid (12, 14). However, there are still many metabolites that have not been studied. We used non-targeted metabolomics techniques to detect fundamental alterations in the serum metabolite profile after inulin supplementation. According to the screening criteria, a total of 113 differential metabolites were screened. Interestingly, the change in indole-3-acrylic acid level was the most pronounced among all differential metabolites. Its level was significantly increased by more than 500-fold compared with the OND group, and it had the largest VIP value from PLS-DA. Indole-3-acrylic acid is a tryptophan metabolite secreted by gut microbiota (43, 44). A study has shown that tryptophan can be converted to indole pyruvate acid (IPyA) by the aromatic amino acid aminotransferase from *Lactobacilli*, while IPyA is the critical precursor of indole-3-acrylic acid (44). Indole-3-acrylic acid is closely related to human health. A study has shown that several *Peptostreptococcus* species could produce the tryptophan metabolite indole-3-acrylic acid, promoting intestinal epithelial barrier function and reducing inflammation. It has a specific therapeutic effect in patients with inflammatory bowel disease (IBD) (43). Metabolites derived from gut microbes (e.g., indole-3-acrylic acid) may attenuate atherosclerosis development in ApoE-deficient rats (45). In addition, the gut microbiota-related metabolite indole-3-acrylic acid was also associated with immune-related diseases (46). There are few studies on the relationship between indole-3-acrylic acid and obesity. In our current study, indole-3-acrylic acid exhibited a strong negative correlation with body weight.

Significant changes in gut microbiota and serum metabolites were observed after 10 weeks of inulin supplementation in obese mice compared to the OND group. Both *Alistipes* and indole-3-acrylic acid showed a strong positive correlation with inulin. The gut microbiota exerts most of its physiological roles mainly through various metabolites (47–50). A study showed that the gut microbiota tryptophan metabolite indole-3 carboxylic acid could regulate energy expenditure and insulin sensitivity by regulating the expression of miRNA-181 in white fat and thus affect obesity (51). Another study found that alternate-day fasting could change gut microbiota composition in mice, increase the synthesis of acetic acid and lactic acid, and induce browning of white adipose tissue (52). Secondary bile acids, metabolites of gut microbiota, are involved in the regulation of glucose and lipid metabolism, and can also enhance insulin sensitivity and promote fat metabolism, which is closely related to the formation of obesity and diabetes (53, 54). Changes in serum metabolites may be mediated by altered gut microbiota after inulin supplementation. Spearman correlation analysis showed that *Alistipes* exhibited a strong positive correlation with indole-3-acrylic acid. According to the taxonomic database of the US National Center for Biotechnology Information, the genus *Alistipes* consists of 13 species, 7 of which can catalyze the production of indole from tryptophan, including *Alistipes finegoldii*, *A. onderdonkii*, *A. shahii*, *A. senegalensis*, *A. timonensis*, *A. putredinis*, and *A. inops* (31). In our study, the *Alistipes* were significantly negatively correlated with tryptophan biosynthesis pathway. The level of tryptophan was reduced considerably after inulin supplementation, which may be due to the rapid metabolism of tryptophan by *Alistipes* to produce a large amount of indole-3-acrylic acid. But further experiments are needed to prove the relationship between *Alistipes* and indole-3-acrylic and to clarify which species of *Alistipes* genus produces indole-3-acrylic acid.

## Conclusion

In the present study, inulin supplementation reversed the changes in the richness, composition, and diversity of gut microbiota induced by HFD. The abundance of the genus *Alistipes* and the level of indole-3-acrylic acid were considerably increased in response to inulin supplementation. Both *Alistipes* and indole-3-acrylic acid were involved in tryptophan metabolism, meanwhile, they were also positively related to inulin supplementation and negatively related to obesity. These findings suggest that inulin accelerates weight loss in obese mice, possibly due partly to increased levels of *Alistipes* and indole-3-acrylic acid. However, the interrelationship between *Alistipes* and indole-3-acrylic acid still need further experiments to prove.

## Data availability statement

The original contributions presented in this study are publicly available. This data can be found here: http://www.ncbi.nlm.nih.gov/bioproject/877788, accession number: PRJNA877788.

## Ethics statement

This animal study was reviewed and approved by the Ethical Committee of Shihezi University (No. A2020-001-01).

## Author contributions

ZW and HW: experimental design, data processing, graphical visualization, and manuscript writing and editing. ZW, ZD, YT, ML, KZ, and YZ: project administration, animal experiment and sample collection. All authors contributed to the work presented in this article.

## Footnotes

1
www.cloudtutu.com

## Supplementary material

Supplementary Figure 1 The average food consumption in grams of each group per week.

Supplementary Figure 2 Correlation analysis between characteristic gut microbiota **(A–C)**.

Supplementary Figure 3 Cladogram generated from LEfSe analysis showing the relationship among taxons **(A,B)**.
