# Supplementary figures and images for: Inulin accelerates weight loss in obese mice by regulating gut microbiota and serum metabolites
Source: Front Nutr. 2022 Sep 28;9:980382. doi: 10.3389/fnut.2022.980382 (PMC9554005; doi:10.3389/fnut.2022.980382)

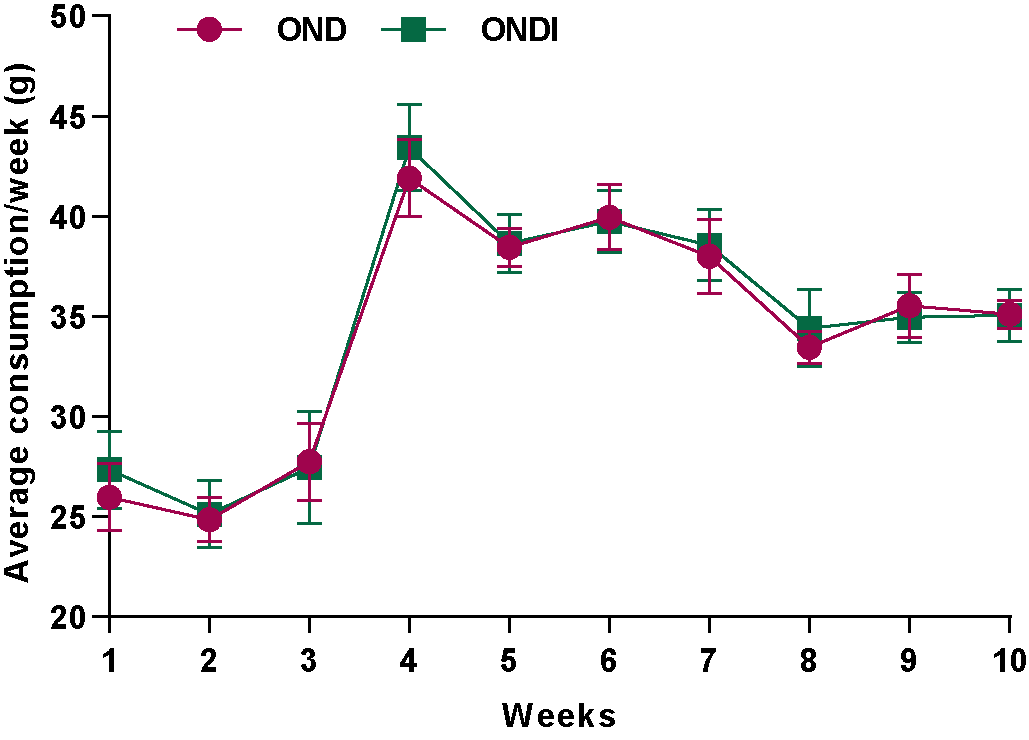

Supplement: Supplementary Figure 1 — The average food consumption in grams of each group per week. [file Image_1.tif]

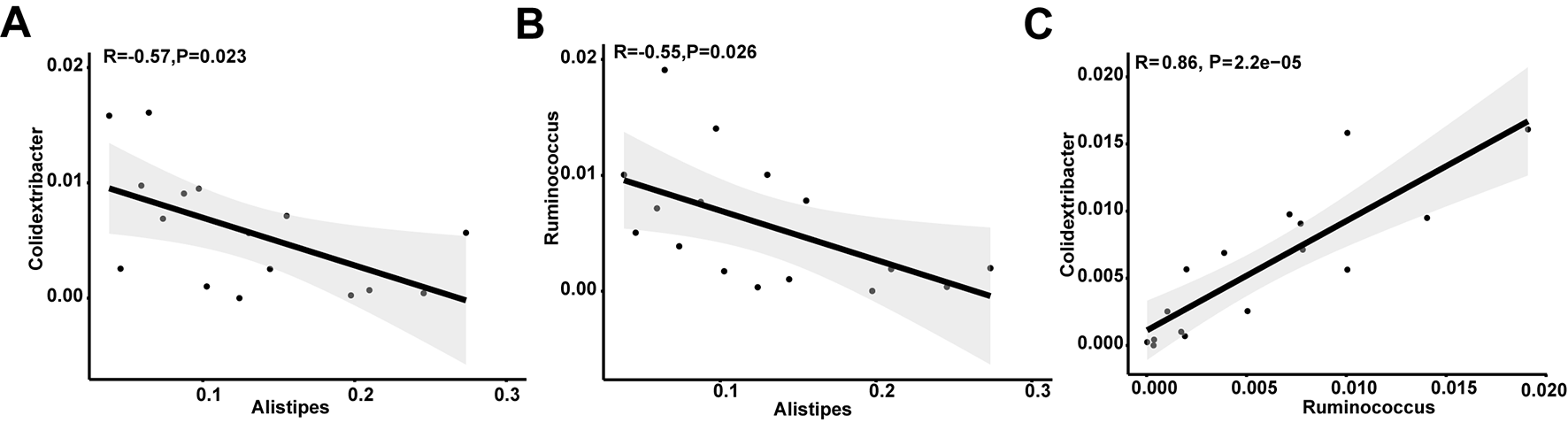

Supplement: Supplementary Figure 2 — Correlation analysis between characteristic gut microbiota (A–C). [file Image_2.tif]

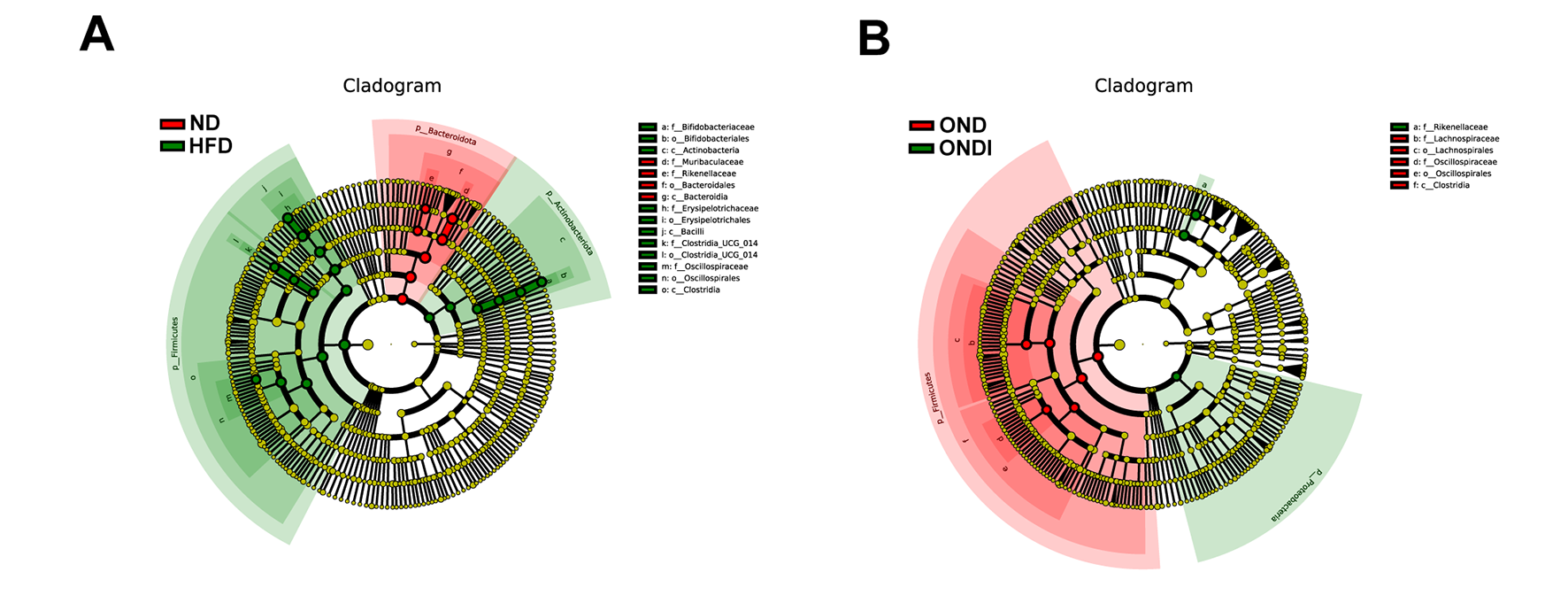

Supplement: Supplementary Figure 3 — Cladogram generated from LEfSe analysis showing the relationship among taxons (A,B). [file Image_3.tif]
